# The PB1 gene from H9N2 avian influenza virus showed high compatibility and increased mutation rate after reassorting with a human H1N1 influenza virus

**DOI:** 10.1186/s12985-022-01745-x

**Published:** 2022-01-25

**Authors:** Hongrui Cui, Guangsheng Che, Mart C. M. de Jong, Xuesong Li, Qinfang Liu, Jianmei Yang, Qiaoyang Teng, Zejun Li, Nancy Beerens

**Affiliations:** 1 Shanghai Veterinary Research Institute, Chinese Academy of Agriculture Sciences, Office Room D301, Ziyue Road 518, Minhang District, Shanghai, 200241 China; 2 Quantitative Veterinary Epidemiology, Animal Sciences Group, Wageningen University and Research, Droevendaalsesteeg 1, 6708PB Wageningen, The Netherlands; 3 Wageningen Bioveterinary Research, Wageningen University and Research, Houtribweg 39, 8221RA Lelystad, The Netherlands

**Keywords:** Reassortment, Human influenza, Avian influenza, H9N2, Mutation rate, NGS

## Abstract

**Background:**

Reassortment between human and avian influenza viruses (AIV) may result in novel viruses with new characteristics that may threaten human health when causing the next flu pandemic. A particular risk may be posed by avian influenza viruses of subtype H9N2 that are currently massively circulating in domestic poultry in Asia and have been shown to infect humans. In this study, we investigate the characteristics and compatibility of a human H1N1 virus with avian H9N2 derived genes.

**Methods:**

The polymerase activity of the viral ribonucleoprotein (RNP) complex as combinations of polymerase-related gene segments derived from different reassortment events was tested in luciferase reporter assays. Reassortant viruses were generated by reverse genetics. Gene segments of the human WSN-H1N1 virus (A/WSN/1933) were replaced by gene segments of the avian A2093-H9N2 virus (A/chicken/Jiangsu/A2093/2011), which were both the Hemagglutinin (HA) and Neuraminidase (NA) gene segments in combination with one of the genes involved in the RNP complex (either PB2, PB1, PA or NP). The growth kinetics and virulence of reassortant viruses were tested on cell lines and mice. The reassortant viruses were then passaged for five generations in MDCK cells and mice lungs. The HA gene of progeny viruses from different passaging paths was analyzed using Next-Generation Sequencing (NGS).

**Results:**

We discovered that the avian PB1 gene of H9N2 increased the polymerase activity of the RNP complex in backbone of H1N1. Reassortant viruses were able to replicate in MDCK and DF1 cells and mice. Analysis of the NGS data showed a higher substitution rate for the PB1-reassortant virus. In particular, for the PB1-reassortant virus, increased virulence for mice was measured by increased body weight loss after infection in mice.

**Conclusions:**

The higher polymerase activity and increased mutation frequency measured for the PB1-reassortant virus suggests that the avian PB1 gene of H9N2 may drive the evolution and adaptation of reassortant viruses to the human host. This study provides novel insights in the characteristics of viruses that may arise by reassortment of human and avian influenza viruses. Surveillance for infections with H9N2 viruses and the emergence of the reassortant viruses in humans is important for pandemic preparedness.

**Supplementary Information:**

The online version contains supplementary material available at 10.1186/s12985-022-01745-x.

## Background

Highly pathogenicity avian influenza viruses (HPAIVs), specifically the H7 and H5 subtypes, are a continues threat to the poultry industry, as these viruses can cause up to 100% mortality in poultry. In contrast, low pathogenicity avian influenza viruses (LPAIVs) typically cause only mild or no clinical symptoms in poultry. Since the first identification of H9N2 isolated from quail in 1988 [1], H9N2 AIV have been isolated from poultry [2–5] and wild birds [6, 7] across Europe, Asia and North America. Since the 1990s, the wide-spread dissemination of H9N2 AIV in poultry had led to human infections in China [8, 9], Bangladesh [10], Pakistan [11] and Oman [12]. Three stable poultry lineages are recognized with the representative viruses, A/quail/Hong Kong/G1/1997 (G1), A/chicken/Beijing/1/94 (BJ94, also known variously as the Y280 or G9 lineage) and A/chicken/Hong Kong/Y439/1997 (Y439, also known as the Korean lineage) [13]. In 1998, the first human infection with H9N2 AIV was reported in Hong Kong [9], and in the same year, five human patients were confirmed infected with H9N2 AIV in Southern China [14]. Since that, a total of 70 laboratory-confirmed human cases were reported in southern China, other Asian countries and Africa based on the records of WHO till December 2020 [8, 15–17]. The clinical human cases with H9N2 AIV indicated that certain strain, e.g. G1 and BJ94 (Y280 or G9) have a preference for the human-like α-2,6-linked sialic acid (SA) receptor due to some specific gene mutations in the HA (Hemagglutinin) gene [8, 18, 19]. Further evolution of the H9N2 AIV may lead to adaptation of the virus to humans; however, no human-to-human transmission has been reported yet [20].

The co-circulation may enhance the evolution of H9N2 AIV with other LPAI or HPAI viruses in poultry due to reassortment events in which gene segments are exchanged between viruses. Evidence for this is provided by phylogenetic analysis of the (A/Quail/Hong Kong/G1/97) G1-like H9N2 AIV [21]. Reassortment events, in particular for the PB2 (Polymerase basic protein 2), HA, NP (Nucleoprotein), and NA (Neuraminidase) genes, resulted in rapid evolution of the AIV, either assist the transmission from the wild bird viruses to the poultry or to humans, or influence its pathogenicity [22–26]. As H9N2 AIVs were shown to have the potential to infect humans, reassortment with seasonal human influenza viruses may also occur during co-infections. The exchange of gene segments between different viral strains can cause sudden changes in pathogenicity, virulence or transmission ability [27–29]. This can give rise to novel influenza viruses that are adapted to the human host. Experimental studies using a ferret model showed that reassortant viruses harboring H9N2 AIV surface genes and seasonal human H3N2 influenza virus internal genes (genes, PB2, PB1, PA, NP, M, NS, express proteins not at the surface of the virus) are efficiently transmitted after adaptation by serial passaging [30]. In addition, human infections were reported with other zoonotic subtypes of avian influenza, such as H5Nx, H7N9, and H10N8 [31]. Genetic analysis of these zoonotic viruses revealed that they arose by re-assortment events with H9N2 AIVs, in which they obtained internal genes of H9N2 AIVs [32, 33]. Several mutations were identified in internal genes of avian H9N2 strains that enhance transmission from poultry to mammals or increase virulence of the viruses [34, 35], such as the mutation 627E/701D in the PB2 gene.

Beside re-assortment events, the high mutation rate of influenza viruses also contributes to their fast evolution. Replication of the RNA genome is mediated by the virus encoded RNA polymerase, which is highly error prone. This results in virus population, or quasi-species, with high genetic diversity. This diversity allows a viral population to rapidly adapt to dynamic environments, thereby allowing escape from the host immune response or vaccination. Particular genetic substitutions in the two surface proteins, HA and NA, can lead to changes in the antigenicity of the virus [36]. The viral RNA polymerase complex is composed of the PB2, PB1 (Polymerase basic protein 1) and PA (Polymerase acidic protein) [37], packaged by the NP protein [38] to generate the viral ribonucleoprotein complexes (RNPs). The RNPs provide the minimal set of proteins required for transcription and replication of viral RNAs [39, 40]. The polymerase proteins were previously reported to affect replication and virulence of influenza viruses [41]. The PB2 protein was found to influence virulence [42, 43] as well as host-preference [44, 45]. A study on single gene reassortment identified a critical role for PB1 (in addition to HA and NA) gene in the high virulence of the 1918 pandemic influenza virus (H1N1 virus) [46]. It was also suggested that the PA gene of (pandemic) H1N1/2009 origin might be contribute to an increased pathogenicity in the reassortants with avian H9N2 strain [26]. Reassortment events between human and avian influenza viruses in combination with rapid evolution and adaptation due to error-prone replication may lead to a novel pandemic. In this study, we investigate the replication capacity of the human WSN-H1N1 virus in combination with avian polymerase genes derived from the A2093-H9N2 virus. We show that the PB1 gene of the H9N2 AIV is able to increase polymerase activity and error-rate of the reassortant human viruses. The avian PB1 gene thus may thereby drive the evolution of novel human reassortant viruses. As the H9N2 AIV is currently still circulating intensively in poultry populations in Asia, and human H9N2 infections have been observed, the emergence of novel reassortant viruses in humans must be carefully monitored for pandemic preparedness.

## Methods

### Viruses and reverse genetic platform

A laboratory mouse-adapted human IAV strain, A/WSN/1933 (H1N1, GenBank: LC333185.1) and an avian-adapted IAV strain, A/chicken/Jiangsu/A2093/2011, (H9N2, GenBank: KP865958.1) were used in this research. The reverse genetic systems for WSN (A/WSN/1933) and A2093 (A/chicken/Jiangsu/A2093/2011) [47] have been constructed using the pBD bidirectional expression vector. The viruses were generated by reverse genetics as previously reported [48, 49]. The reverse genetics system was used to generate stable-replicating reassortant viruses containing segments of A2093-H9N2 in the WSN-H1N1 virus background. One of the internal genes of WSN/H1N1 (PB2, PB1, PA or NP) and the surface genes (both HA and NA) were replaced by that of the avian A2093-H9N2 virus to obtain PB2-reassortant, PB1-reassortant, PA-reassortant and NP-reassortant. The wild type (wt) A2093-H9N2 and WSN-H1N1 were also rescued in this system for control.

### Cell lines and animals

Madin-Darby Canine Kidney (MDCK) cells and avian DF-1 cells were cultured in Dulbecco's modified Eagle's medium (DMEM, high glucose Gibco™) containing 5% fetal bovine serum (FBS, Gibco™) and 1% penicillin–streptomycin. The 293T cells were cultured with DMEM containing 10% FBS and 2 mM L-glutamine (Gibco™). All cell lines were incubated at 37 ℃ in humidified incubator with 5% CO_2_.

Specific-pathogen-free (SPF) 4- to 6-week-old female BALB/c mice were purchased from Vital River Laboratories, Beijing, China. They were housed in isolators with air re-circulation system. The room housing isolators was controlled by the central air conditioning to 23–25 ℃ with 40–60% humidity. Mice were housed under optimal light conditions and feed and water were provided ad libitum. Sawdust and corn cob beddings were provided in the isolators. Bedding materials, food and water were refreshed every week. Virus infection experiments were carried out in high containment facilities, each isolator had a separate air-circulation purification system. The mice were euthanized at the end of the experiment. Manure was removed at the end of the experiment.

### Activity of the reassortant polymerase in vitro

A minigenome assay was performed to compare the activities of viral RNP complexes with one of the polymerase-related gene from avian influenza H9N2 (A2093) following the manufacturer’s instructions of Dual-Luciferase Reporter 1000 Assay Systems (Promega Corporation, Wisconsin, USA). Briefly, we constructed 8 eukaryotic expression plasmids with genes from virus strains A2093-H9N2 and WSN-H1N1, in the backbone of the pCAGGS expression construct: A2093-PB2, A2093-PB1, A2093-PA, and A2093-NP and WSN-PB2, WSN-PB1, WSN-PA, and WSN-NP.

Then 1 × 10^5^ 293T cells were cultured in 12-well plates together with the four protein expression plasmids (pCAGGS PB2, pCAGGS PB1, pCAGGS PA, and pCAGGS NP [0.5 g of each]) for each of the 8 RNP combinations of A2093-H9N2 and WSN-H1N1 virus proteins. The open reading frame (ORF) of Firefly Luciferase was inserted in the packaging signals of NA gene (N′ 183 base pairs (bp) and C′ 157 bp) of the PR8 strain (A/Puerto Rico/8/1934), was placed under control of the pPol I promoter, and was inserted into a multiple cloning site (MCS) of pUC18 plasmid (Takara Bio, Dalian, China). At 48 h post transfection, the Relative light units (RLU) of Firefly Luciferase were measured on a GloMax 96 microplate luminometer (Promega) according to the manufacturer’s instructions. As an internal control for the dual-luciferase assay, Renilla (20 ng each, Promega) was used. The results were presented as the mean and standard deviation of three independent parallel experiments.

### Replication of H1N1/H9N2 reassortant viruses in MDCK cells and mice

The growth kinetics of the rescued viruses were estimated by infection of mammalian MDCK cells, and avian DF1 cells at 0.001 multiplicity of infection (MOI). The 70%-80% monolayer cells were prepared in three T25 flasks 12 h before infection. One flask of cells was used for counting the number of cells. The other two are for parallel control. A total of 1 ml virus solution was incubated on the cells for 1 h at 37 ℃, 5% CO_2_ incubator. After removing the virus solution, 5 ml fresh SFM (Serum-Free Cell Culture, Gibco) was added. Cell supernatant samples (300 µl in volume) were collected at 12, 24, 36, 48, 60 and 72 h post infection to determine the virus titer.

The replication of the reassortant viruses in mice were estimated. For each infection group, three 4-week-old female BALB/c mice were infected intranasally with 10^6^ PFU virus stocks in 50 µl PBS (Phosphate-buffered saline). These mice were sacrificed for the tracheal and lung tissues at 4 d.p.i. Clear supernatants of homogenates were used to evaluate the viral titer. Viral titers of these samples were determined in plaque-forming units per milliliter (PFU/ml) by plaque assays on MDCK cells as previously described [50]. Here we applied immunohistochemical staining method with influenza A virus nucleoprotein monoclonal antibody (clone FluA-NP 2C9) to count the plaques. Three independent experiments were carried out. Mean with standard error (SD) were used for data analysis.

Based on the replication ability and viral titer of parent virus strains, virulence of the virus were further evaluated by observing the body weight loss of infected mice. For each infection group, five 4-week-old female BALB/c mice were infected intranasally with 50 µl 10^6^ PFU virus stocks in PBS (Phosphate-buffered saline). As control, five 4-week-old female BALB/c mice were inoculated with same amount of PBS. Five mice were numbered, the body weight and healthy condition were observed and record every 24 h for 14 days post infection (d.p.i). The percentage of the body weight change after infection was calculated comparing to the original body weight measured before infection (0 d.p.i). Average percentage body weight loss of five mice of every group were calculated. Multiple t-test with a = 0.05 were performed for significant differences.

### Serial passaging of H1N1/H9N2 reassortant viruses in cells and mice

Reassortant viruses, wt WSN-H1N1 and A2093-H9N2 viruses were passaged in MDCK cells at a MOI of 0.001. In detail, three T25-flasks of 80% monolayer cells were seeded 12 h before the infection. Parent virus was inoculated with cells at a MOI of 0.001 for one hour at 37 ℃, 5% CO_2_. Cells were washed three times with SFM (Thermo Fisher Scientific) and then maintained with SFM for at least 24 h at 37 ℃, 5% CO_2_. The HA titer of supernatant and cell conditions were checked every 6 h till the HA titer was greater than 16 HA Units and more than 70% cell lysis was observed. The progeny viruses were incubated for a new generation host-circle with the same amount of MDCK cells right after collected from the previous generation.

In vivo, reassortant WSN-H1N1/A2093-H9N2 viruses and the corresponding wt WSN-H1N1 and A2093-H9N2) were passaged in 4-week-old BALB/c mice. Three mice were intranasally inoculated with 10^6^ PFU parent virus stocks and sacrificed for the tracheal and lung tissues at 4 d.p.i. The homogenates of the two tissues from 3 mice were mixed separately, and subsequently used for infection (intranasally) of two groups of mice. One group as for serial passaging in mouse lung (3 mice), and the other group for the serial passaging in mouse trachea (3 mice). During the serial passaging, the age and health condition of the mice were maintained the same as possible.

The above serial passaging procedures were repeated five times for five generations, and two independent parallel passaging experiments were performed.

### Sample preparation for NGS

The reassortant WSN-H1N1/A2093-H9N2 viruses and the wt strains obtained after serially passaging in MDCK cells and mice (lung and turbinate, specifically) were prepared for Next Generation Sequencing (NGS). The initial virus stock and the last generation (P5) were selected for NGS sequencing. The details of sequenced viruses are listed in Additional file 2: Table S1. All samples were analyzed in duplicate, starting from two independent RNA isolations.

Viral RNA (vRNA) was extracted from 140 μl supernatants from cell passaging or tissues’ homogenates by using QIAGEN Viral RNA Isolation Kit following the manufacturer’s instructions. The isolated RNAs were all eluted into 30 μl diethylpyrocarbonate-treated water. Two-step RT-PCR was employed to amplify each viral gene segment. The first-strand cDNA was transcripted by using Transcriptor High Fidelity cDNA Synthesis Kit (ROCHE) with universal primer (5′-AGCAAAAGCAGG-3′) for influenza A virus in a final volume of 20 μl as manufacturer’s protocol. The HA gene was amplified using primer-pairs to obtain amplicons of approximately 500bp per PCR run. Accordingly, there were four fragments of HA gene, two fragments of M and NS genes respectively. Six-nucleotide indexes were added to the forward primers for PCR to allow bar-coding of the samples (Additional file 2: Table S1). All the primers for PCR ware listed in Additional file 2: Table S2. In detail, the PCR amplification was carried out with Phanta Max Super-Fidelity DNA Polymerase (Vazyme Biotech Co.,Ltd) in a 50 µl system, using 2 µl cDNA template. The amplification program consisted of a 3-min period at 95 ℃ and was followed by 35 cycles with the following conditions: 95 ℃ for 30 seconds, 55 ℃ for 30 s, and 72 ℃ for 40 seconds, and ended with one cycle at 72 ℃ for 5 min. The amplicons were purified in 1% agarose gel electrophoresis and then purified using a DNA Gel Extraction Kit (Axygen, Hangzhou, China). The concentration and quality were estimated by using NanoDrop 2000C. The concentration ranges of the PCR products were 15–150 ng/ul, and the 260/280 absorption was between 1.6 and 2.0. All bar-coded PCR products of the same gene fragment were mixed in equal quantities (30 ng) to construct one DNA library for NGS analysis. The DNA libraries were generated using VAHTS Universal DNA Library Prep Kit for Illumina.

### Analysis of NGS data

The libraries were sequenced using Illumina MiSeq at 2 × 150 bp (4.5 Gb for 10–15 M read pairs) configuration (Illumina, San Diego, CA, United States) at high coverage (average > 10,000 per nucleotide position) by GENEWIZ (Suzhou, China). Quality control-passed sequence reads were mapped using the WSN and A2093 virus reference sequences, and used to detect minority variants. The primer sequences were removed from the reads, then overlapping region of two PCR fragments was avoided by mapping the reads till the median location. From the mapped data, we obtained the reads counts (sequencing depth) of each nucleotide type (A/T/C/G) at every location of the whole length of HA gene, N_ij_ (*i* is the position of the substitution on the gene; *j* refers to one nucleotide type, A/T/C/G). Then the frequency of each nucleotide type at the location can be approached as f_ij_ = N_ij_/ C_i_ (C_i_ is the sequencing depth at location i). Based on the reference sequence, total substitution frequency at every location was available as ${\text{f}}_{{\text{i}}} = \sum\nolimits_{j}^{3} {f_{ij} }$ (Additional file 2: Table S3). The sequencing errors, that occurred at every location of the gene, are identically independent distributed with a binomial distribution. And the probability of observing a substitution with the sequencing errors as background nioce follows approximately a Poisson distribution. To set up the threshold of the background noice and the dicersity in parent virus population, webinned the f_i_ valuses of sequencing data from parent A2093 strain with a range of 0.001. There were 95% f_i_ valuses in bins before the bin of range 0.005–0.006. Therefore, f_i_ $\ge$ 0.6% were filtered as minority single base substitutions (SBS). Geometric mean of minority SBS indicating the mutation rate of these “hot-spots” on HA gene. This was calculated by the geometric mean ${\text{F}}_{{\text{g}}} = \left( {\sum\nolimits_{i}^{n} {f_{i} } } \right)^{\frac{1}{n}}$, *n* represented the nucleotide number of the locations with ${\text{f}}_{{\text{i}}} \ge 0.6$%. However, the general mutation rate over a certain domain of the HA gene (F_g_ per nt) was calculated by the arithmetic mean F_g_ per ${\text{nt}} = \frac{1}{N}\sum\nolimits_{i}^{N} {f_{i} }$, N was the nucleotide numbers of the sequence region of HA gene. The genetic distance of nucleotide sequence between two virus stock A and B was calculated with a population wide measure ${\text{d}} = \sqrt {\frac{1}{N}\sum\nolimits_{i}^{n} {\left( {f_{iA} - f_{iB} } \right)^{2} } }$ [51], N was the nucleotide numbers of the sequence region of HA gene. In this research, we calculated the distance of reassortant viruses (f_iA_) to that of parent A2093-H9N2 (f_iB_). Calculations were programmed in R Studio [52].

For the calculation of transition/transversion and non-synonymous mutations, we extended the threshold of f_i_ to $\ge$ 1%, as detection limit for reliable recognition of variants in the viral [53] and the background error from PCR and sequencing. For transition/transversion definition, one SBS with f_i_ $\ge { }$ 0.01 observed at one location was counted as one occurrence of transition (Ts) or transversion (Tv) basing on the consensus sequence. Sum of all the f_i_ of transition or transversion over the whole genome was the probability that transition and transversion observed after selection, as P_Ts_ and P_Tv_. Simultaneously, the total numbers of Ts and Tv were summed up, which indicating the diversity of one viral population (one virus stock).

Data in detail are displayed in Additional file 2: Table S4. Two parallel independent NGS runs (run1, run2) were performed to analyze the reproducibility of the results. NGS data from these two runs of sequencing were compared. Multiple t-test Discovery was applied using the Two-stage linear step-up procedure of Benjamini, Krieger and Yekutieli, with Q = 1%. Each row was analyzed individually, without assuming a consistent SD by using GraphPad Prism.

## Results

### Polymerase activity of reassortant viruses in vitro

**Fig. 1:**
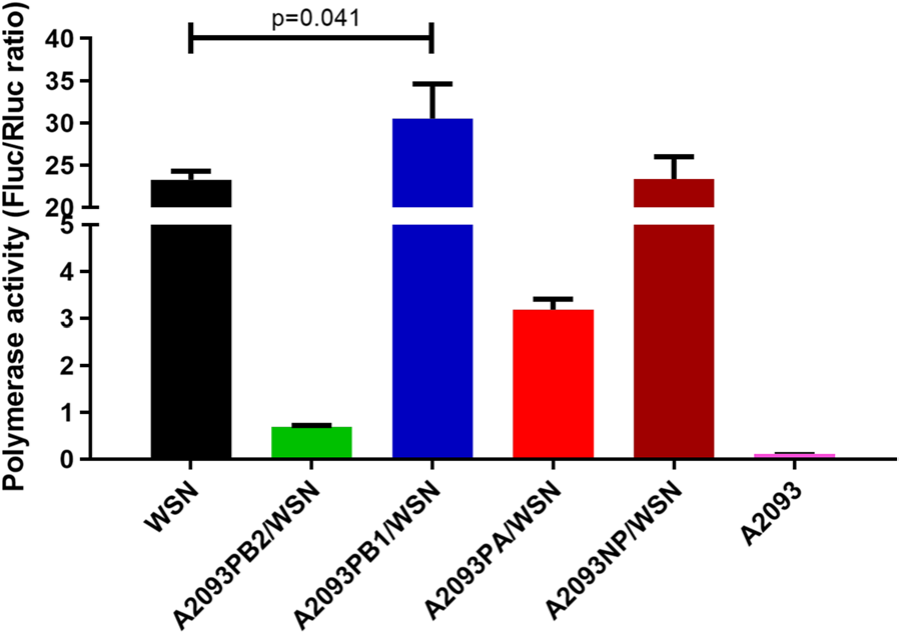
Viral RNPs functional assay of recombinant polymerase complex. The polymerase activities of the human WSN-H1N1 RNP, the recombinant RNPs containing PB2, PB1, PA or NP genes derived from the avian A2093-H9N2 strain, and the avian A2093-H9N2 RNP were measured using the Dual-Luciferase Assay System. Firefly luciferase activity was measured to determine polymerase activity, and Renilla luciferase activity was used as an internal control for monitoring transfection efficiency. The Y axis displayed the Fluc (firefly) /Rluc (Renilla) ratios. Experiments were repeated three times, mean and SD were applied. Multiple t-test with a = 0.05. *significant difference

The polymerase activity of the avian A2093-H9N2 influenza virus was compared to that of the human WSN-H1N1 influenza virus using an in vitro Luciferase reporter assay. In addition, reassortant H1N1 and H9N2 polymerases were tested in which either the PB2, PB1, PA or NP genes were exchanged between the two viruses. The activity of the recombined polymerases was compared to that of the WSN-H1N1 and A2093-H9N2 polymerases (Fig. 1). Compared to the WSN-H1N1 polymerase, the reassortant polymerase containing the PB1 gene of the avian A2093-H9N2 virus significantly increased the activity of polymerase complex in vitro (mean diff. − 7.245, 95% CI of diff. − 12.76 to − 1.734). Replacement of the PB2 or PA genes dramatically decreased the polymerase activity (mean diff. were 22.57 and 20.07 respectively), whereas replacement of NP did not significantly affect the activity (mean diff. − 0.09848, 95% CI of diff. − 5.610 to 5.413).

### Replication of the reassortant viruses in cells

**Fig. 2:**
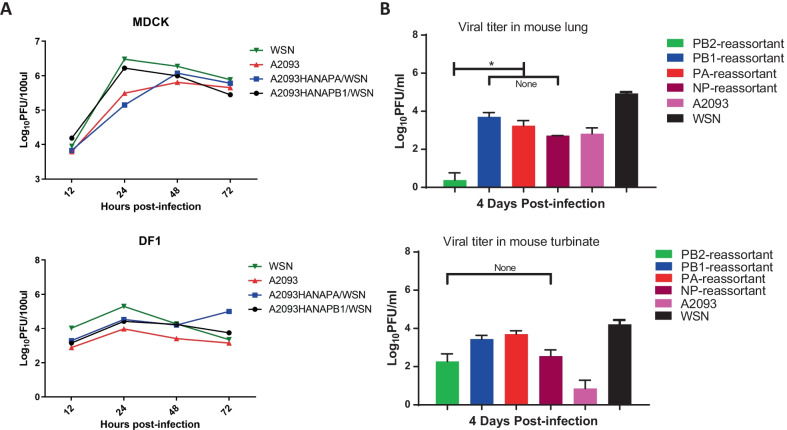
Replication of reassortant viruses in cell lines (**A**) and in mouse (**B**). **A** Virus replication of human wt WSN1-H1N1, avian wt 2093-H9N2 and reassortant viruses containing the avian PB2, PB1, PA or NP segments. Virus replication was measured by determining plaque forming units at several hours post infection of MDCK and DF1 cells. **B** Virus replication of human wt WSN1-H1N1, avian wt 2093-H9N2 and reassortant viruses containing the avian PB2, PB1, PA or NP segments. Virus replication in mouse turbinal tissue and lung was measured at. 4 days post infection using plaque assays. Plaque assays were repeated three times, the mean and sd of the PFU in logarithm of 10 was applied. Multiple *t* test with a = 0.05. *Significant difference. None indicated no significant difference

Reassortant WSN-H1N1 viruses were generated, in which both the HA and NA genes were replaced by that of A2093-H9N2, in combination with one of the polymerase-related genes PB2, PB1, PA or NP. The replication of these viruses was studied after infection of mammalian MDCK cell and duck DF1 cells, as shown in Fig. 2A. The viral titers of wild-type and reassortant viruses were evaluated using plaque assays and HA titers were determined (Table 1). In MDCK cells, the replication of the PB1-reassortant viruses was similar to that of wt WSN-H1N1 virus. The peak viral titers were obtained after 24 h, 2.83 × 10^6^ (± 1.17 × 10^6^) PFU/ml for wt WSN-H1N1, and 5.69 × 10^6^ (± 1.10 × 10^6^) PFU/ml for the PB1-reassortant virus. For the other reassortant viruses, delayed replication kinetic peak and lower titers were observed: 2.21 × 10^4^ (± 1.23 × 10^4^) PFU/ml for PB2-reassortant to 1.28 × 10^6^ (± 1.25 × 10^6^) PFU/ml for PA-reassortant. On DF1 cells titers of around 10^2^–10^5^ PFU/ml were obtained for the reassortant viruses, whereas a peak titer of 2.09 (± 1.2) × 10^5^ PFU/ml was observed for wt WSN-H1N1 virus at 24 hpi. On DF1 cells, lower peak viral titers were observed compared to MDCK cells. Due to inefficient replication on DF1 cells, MDCK cells were selected for subsequent serial passaging experiments with the reassortant viruses.

**Table 1:** The viral titers of viruses before and after 5-generation's serial passaging

Rescued viruses	Parent Virus HA titer (log2)	Parent Virus (generation 0) (log10 ^copies^/ml)^a^	5th Generation from MDCK cell line (log10 copies/ml)	5th Generation from Mouse lung (log10 copies/ml)
A2093HANAPB2/WSN	10	8.05 ± 0.02	2.16 ± 0.17	2.56 ± 0.82
A2093HANAPB1/WSN	6	7.09 ± 0.04	7.59 ± 0.01	6.59 ± 0.48
A2093HANAPA/WSN	10	8.33 ± 0.06	5.48 ± 0.00	7.31 ± 0.28
A2093HANANP/WSN	9	8.06 ± 0.20	2.33 ± 0.05	6.73 ± 0.27
A2093	11	7.76 ± 0.02	6.60 ± 0.00	6.16 ± 0.03
WSN	7	7.87 ± 0.04	6.74 ± 0.00	7.86 ± 0.30

^a^Q-PCR were repeated twice, mean and sd were calculated to estimate the viral particles. It was suggested that copy number of viral particles were 20–60 times of one infectious virus[54]

### Replication and virulence of reassortant viruses in mice

Virus replication for the reassortant viruses was studied by infection of mice. At four days post infection, virus titers in the mouse lung and nasal turbinate were measured (Fig. 2B). The human-originated WSN-H1N1 virus replicated more efficiently in mice than the avian A2093-H9N2 virus, likely due to its adaptation to replication in mammals. The replication of the reassortant viruses containing either the A2093-H9N2 PB2 or NP genes was reduced compared to wt WSN-H1N1 virus. However, virus replication of the WSN-H1N1 was not significantly affected by replacement with the avian PB1 and PA genes. That is, these two reassortant viruses were able to replicate in both turbinate and lung up to 10^4^ PFU/ml. The A2093-H9N2 showed a slight preference for replication in the lung compared to turbinate. The reassortant viruses were able to replicate to titers of 10^3^ PFU/ml (without significant differences between the viruses), except for PB2-reassortant virus for which significantly lower titers were obtained. These results show that lowest levels of replication were observed for the PB2-reassortant virus in both cell lines and mice, whereas the other reassortant viruses replicate to similar levels.

**Fig. 3:**
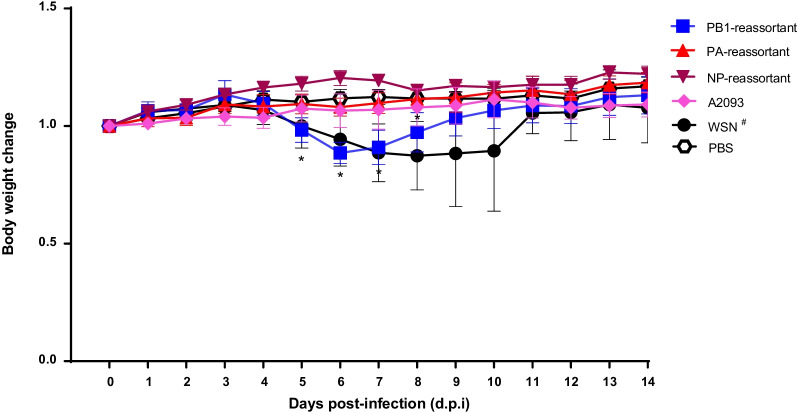
Virulence of reassortant viruses in mice. Virulence of the PB1-, PA- and NP-reassortant viruses was measured by infection of five mice, after which body-weight loss was measured for 14 days after infection. For reference, the human wt WSN-H1N1 and avian wt A2093-H9N2 viruses are shown. The body weight changes is plotted, and significant differences (Multiple *t* test with a = 0.05) are marked (*). The body weight changes are expressed as the mean with SD in each group (n = 5). ^#^One mouse died on day 8, 9 and 11 d.p.i

To study the virulence of the reassortant viruses, we inoculated 8 mice with the reassortant viruses that were found to efficiently replicate in mice (PB1, PA and NP-reassortant viruses). Subsequently, the body-weight loss of five mice was measured during 14 days after infection as a marker for virulence. This showed that the PB1-reassortant virus caused significant body-weight drop (Fig. 3) in infected mice, similar to the wt WSN-H1H1 virus at the 5th, 6th and 7th d.p.i. After inoculation with the PB1-reassortant virus, significant body-weight loss was observed with decreasing trend on 5th, 6th and slight increasing trend on 7th and 8th d.p.i. The increasing trend of WSN-H1N1 strain was observed after 10 d.p.i. No weight loss was observed for the NP-reassortant and PA-reassortant viruses, however the PA-reassortant virus was detected at low level in lung and turbinate at 4 d.p.i (Fig. 2B). In conclusion, the PB1-reassortant virus was able to replicate in mice and resulted in significant body weight loss.

### Serial passaging of reassortant viruses

To study the replication and evolution of the viruses during prolonged passaging, the reassortant viruses and wt A2093-H9N2 virus were passaged in both MDCK cells and in mice. As a result of low replication for the PB2-reassortant virus in both cell lines and mice, we were unable to obtain the progeny viruses of this reassortant strain. However, five generations of progeny viruses of the PB1, PA and NP-reassortant viruses and wt A2093-H9N2, WSN-H1N1 virus were collected from serial passaging on MDCK cells.

These reassortant viruses and the A2093-H9N2 virus were also successfully passaged for five generations in mice. Viruses harvested from individual mouse lung or turbinate were pooled and inoculated into the next group of mice. We were able to continue passaging for five generations in mice lung, however, for turbinate only one generation was successful.

The viral titers of parent virus before inoculation and the 5th generation of progeny viruses is listed in Table 1. For the following analysis, the PB2-reassortant strain was not included due to unsuccessful serial passaging. There was no significant difference in the titers obtained for the other viruses, suggesting a similar population size for subsequent analysis of viral sequences.

### General mutation frequency and hot-spots on HA gene

**Fig. 4:**
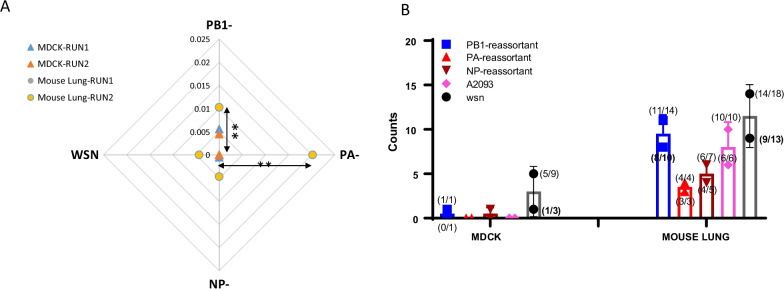
Genetic variation in the HA sequence after passaging of reassortant viruses. **A** Genetic distance of HA sequences. The genetic distance of the HA sequences of the PB1-, PA- and NP-reassortant viruses after five passages in MDCK cells (triangles) and mouse lung (circles) is plotted relative to the wt A2093-H9N2 virus. The genetic distance of the WSN-H1N1 after five passages is plotted relative to the wt WSN-H1N1 virus. Two independent experiments were performed in cells and mice, that were analyzed by NGS and are plotted separately, as RUN1 and RUN2. Further details on the NGS results are shown in Additional file 2: Table S5. ** significant difference in the genetic distance compared to the WSN-H1N1 strain. **B** Non-synonymous numbers over open read frame (ORF) of HA nucleotide sequence. The number of substitutions (SBS) is plotted for the PB1, PA and NP reassortant viruses, and the wt A2093-H9N2 and WSN-H1N1 viruses, after passaging in MDCK cells and mice lung. Numbers in the bracket indicated non-synonymous numbers with F_i_ > 0.01, and the total SBS number separating by “/”

We analyzed the mutation rate of the reassortant viruses during serial passaging in both MDCK cells and mouse lung. The virus population was analyzed by Illumina sequencing, the reads were mapped onto the HA ORF region of the reference sequence. Comparing the mutation rates (F_g/nt_, mutation/site/infection cycle) calculated from the HA1 and HA2 region (Table 2), after five passages in mouse lungs, showed that the net mutation rates (after selection) of the PB1- and PA-reassortant viruses were almost 2–3 times higher than that of A2093-H9N2 and WSN-H1N1 strains. The genetic distance in the HA sequences of the viruses after 5 passages in MDCK cells and mouse lung was calculated (shown in Fig. 4A). This analysis showed that for the PB1- and PA-reassortant viruses, the genetic distance in the HA sequences significantly increased compared to that of the wt A2093-H9N2 virus after passaging in mouse lung. No significant difference was observed for the NP-reassortant after passaging in mouse lung. For none of the viruses a significant difference in the genetic distance was observed after passaging in MDCK cells. The genetic distances of the HA sequences observed during passaging of the PB1- and PA-reassortant viruses in mouse lung were also larger than measured during passaging of the WSN-H1N1 virus (Fig. 4A, details in Additional file 2: Table S5). Differences were observed in the substitution frequency measured for different domains in the HA gene (Table 2). The region from 350 to 950 on HA nucleotide sequence (marked as HA1^b^ in Table 2) includes the 130 helix, RBS and 220 loop domains of HA head. The mutation frequency in this functional HA1 domain was compared to that of the complete HA gene, and that of the HA2 domain (region 1015–1615 for A2093-H9N2, region 1030–1630 for WSN-H1N1). The general mutation rates (F_g/nt_, mutation/site/infection cycle) over the HA1 domain was 2.1 times (Std. Error = 9.425E-02) higher than that over the whole HA domain (in linear regression with F_g/nt_ (HA1^b^) = 2.13 F_g/nt_ (whole HA), R^2^ = 0.967, *p*-value = 3.938e-14). The plot of the dataset containing F_g/nt_ of different HA domains were visualized in R, and exported on Additional file 1: Fig. S1. The general mutation rates over the HA2 domain displayed no linear relationship with that over the whole HA regions (Table 2), and the general mutation rates of the HA2 domain were lower than the HA1 domain for all the progeny viruses. These results suggest that the HA1 region, for with higher substitution frequency was measured, reflects a mutation “hot-spot” on the HA gene. This is likely due to increased selective pressure on this functional HA domain.

**Table 2:** The geometric mean of substitution frequency and general mutation rate of viruses from 5th generation

Virus strain	Origination of the 5th generation	F_g_	F_g/nt_^d^	F_g_	F_g/nt_	F_g_	F_g/nt_
		_HA_^a^_(RUN1, RUN2)_		_HA1_^b^_(RUN1, RUN2)_		_HA2_^c^_(RUN1, RUN2)_	
A2093	MDCK	1.13E−02, 0	3.69E−05, 0	9.38E−03, 0	4.94E−05, 0	1.75E−02, 0	2.91E−05, 0
WSN		1.84E−02, 1.32E−02	4.77E−05, 1.37E−04	2.10E−02, 1.63E−02	3.49E−05, 5.82E−05	1.67E−02, 1.53E−02	9.56E−05, 2.47E−04
A2093HANAPB1/WSN		2.35E−02, 3.55E−02	1.71E−04, 1.16E−04	3.27E−02, 3.55E−02	4.16E−04, 3.14E−04	1.58E−02, 0	2.63E−05, 0
A2093HANAPA/WSN		1.20E−02, 0	3.85E−05, 0	1.03E−02, 0	5.31E−05, 0	1.75E−02, 0	2.91E−05, 0
A2093HANANP/WSN		1.25E−02, na	4.82E−05, na	9.99E−03, na	5.21E−05, na	1.57E−02, 0	2.61E−05, 0
A2093	MOUSE LUNG	1.43E−02, 2.24E−02	2.62E−04, 2.34E−04	1.89E−02, 3.72E−02	6.00E−04, 5.32E−04	8.93E−03, 1.22E−02	9.18E−05, 6.19E−05
WSN		1.38E−02, 1.63E−02	2.90E−04, 2.62E−04	1.64E−02, 2.34E−02	5.75E−04, 4.94E−04	1.17E−02, 1.31E−02	1.45E−04, 1.65E−04
A2093HANAPB1/WSN		1.82E−02, 2.49E−02	5.03E−04, 4.98E−04	1.77E−02, 3.44E−02	9.49E−04, 9.04E−04	2.06E−02, 2.12E−02	3.91E−04, 3.99E−04
A2093HANAPA/WSN		3.96E−02, 2.91E−02	6.17E−04, 6.13E−04	4.91E−02, 2.43E−02	1.34E−03, 1.38E−03	4.66E−02, 3.93E−02	3.06E−04, 2.80E−04
A2093HANANP/WSN		1.30E−02, 1.34E−02	1.60E−04, 1.61E−04	1.35E−02, 1.35E−02	3.36E−04, 3.07E−04	8.27E−03, 9.55E−03	4.15E−05, 5.10E−05

The cutoff value for the mutation rate calculation was fi > 0.006 which was generated based on the NGS data from parent virus as backgroud control. The value 0 indicated no locations has the fi > 0.006; the na. indicated no NGS data available. ^a^the H9 HA1&2 of A2093 is 55–1680 of ORF region, the H1 HA1&2 of WSN is 52–1695; ^b^The hot-spot region are 350–950 for both; ^c^HA2 for WSN strain is 1030–1630, for A 2093 strain is 1015–1615. ^d^Fg/nt, mutation/site/infection cycle

### Mutation preference of reassortant viruses

We analyzed the substitutions observed in the HA ORF in further detail with a threshold of f_i_ ≥ 0.01 for significant SBS after selection. The total number of SBS which reflects the diversity of the gene was calculated, as well as the number of transitions and transversion, and the number of synonymous and non-synonymous mutations (Table 3). Few significant SBS were observed in the HA gene after serial passaging in MDCK cells. The total number of SBS detected in the HA ORF was highest for the PB1-reassortant virus after passaging in mice lung. Therefore, the gene diversity of the HA gene of PB1-reassortant virus was higher than estimates for the other reassortant viruses after five passages in mouse lungs.

**Table 3:** Transition/transversion and non-synonymous mutations of viruses passaged for 5 generations

Virus strain	Origination of the 5th generation	SBS Count		Ts		Tv		P_Ts_		P_Tv_		Ratio of P_Ts_ and P_Tv_		Non-synonymous count		Non-synonymous percentage^a^	
		Run1	Run2	Run1	Run2	Run1	Run2	Run1	Run2	Run1	Run2	Run1	Run2	Run1	Run2	Run1	Run2
A2093	MDCK	0	0	0	0	0	0	0	0	0	0	na	na	0	0	na	na
WSN	MDCK	3	9	3	9	0	0	0.0686	0.199	0	0	na	na	1	5	33.33%	55.56%
A2093HANAPB1/WSN	MDCK	1	1	0	1	1	0	0	0.181	0.225	0	0	na	1	0	100.00%	0.00%
A2093HANAPA/WSN	MDCK	0	0	0	0	0	0	0	0	0	0	na	na	0	0	na	na,
A2093HANANP/WSN	MDCK	2	na	1	na	1	na	0.0174	na	0.492	na	0.035	na	1	na	50.00%	na
A2093	MOUSE LUNG	10	6	5	4	5	2	0.2446	0.228	0.109	0.074	2.249	3.092	10	6	100.00%	100.00%
WSN	MOUSE LUNG	18	13	11	13	7	0	0.33	0.362	0.069	0	4.769	na	14	9	77.78%	69.23%
A2093HANAPB1/WSN	MOUSE LUNG	14	10	7	7	7	3	0.2554	0.249	0.811	0.771	0.315	0.323	11	8	78.57%	80.00%
A2093HANAPA/WSN	MOUSE LUNG	4	3	3	3	1	0	0.9448	0.949	0.014	0	67.14	na	4	3	100.00%	100.00%
A2093HANANP/WSN	MOUSE LUNG	7	5	2	2	5	3	0.0986	0.093	0.092	0.068	1.07	1.375	6	4	85.71%	80.00%

Ts (Tv) indicated the number of transition (transversion); P_Ts_ (P_Tv_) indicated the sum of the substitution frequency of all the valid transition (transversion). The cutoff value of fi > 0.01 was to guarantee the observed transitions or transversions were not from system error of sequencing and PCR progress. The value 0 indicated no locations has the fi > 0.006; na. indicated no NGS data or calculation unavailable. ^a^questionable results of progeny viruses in MDCK passaging line because of the observed numbers of SBS were too less for statistical calculation

Substitution frequency (f_i_) for A (Adenine) towards G (Guanine) was higher than that for other types of substitutions on viruses passaged in both MDCK cells and mouse lung. Only for the PB1-reassortant virus, the estimated possibility of transversions (P_Tv_) was higher than that of transitions (Table 3). There was a positive correlation between a higher transition/transversion ratio and a higher non-synonymous mutations ratio for all reassortant viruses. Most of the significant SBS we observed in mouse lung passages were non-synonymous mutations (Fig. 4B). The probability of SBS resulting in a non-synonymous mutation in HA was significantly higher for the PA- and PB1-reassortant viruses after five passages in mouse lung*.*

## Discussions

Influenza viruses are characterized by rapid mutation caused by error-prone viral RNA-polymerase enzyme on the negative-RNA genome during the replication. In addition, the segmented genome of influenza viruses allows these viruses to obtain novel genetic information by reassortment with other influenza strains. Error-prone RNA polymerase activity may be beneficial for the virus by providing diverse gene mutations that may allow rapid adaptation to a new host. Reassortment may also enable the virus to acquire features from other influenza viruses within short infection circles, thereby possibly rapidly adapted to new host population and becomes endemic or pandemic. Many influenza pandemics in history were caused by reassortant viruses originating from mammalian-adapted viruses that obtained genes from avian influenza viruses. For instance, the H1N1 virus causing the 1918 pandemic was a reassortant virus, containing HA from H1 subtype human-adapted strain and NA and other gene segments from avian influenza viruses [55]. Also the viruses causing the 1957 pandemic [56] and the 2009 pandemic [57, 58] were reassortant viruses.

Considering the pandemic threat of avian influenza viruses, it is important to provide more insight in the characteristics of human influenzas viruses after reassortment with avian influenza viruses. To approach this, we simulated the reassortment of human H1N1 virus (inner genes) and avian H9N2 virus (both HA and NA genes, and one of the polymerase genes). The genotype of the H9N2 strain (A2093) used in this research was B69 with its HA gene clustered in Ck/BJ/1/94-like lineage [59]. Besides, it has been reported with higher avidity for α 2,6- sialic acid. This virus strain showed ability to replicate in mammalian cells and in mice. [60]. The surface genes were derived from the avian H9N2 virus, and the reassortant viruses thus were able to replicate but not adapted to the mammalian receptor as well as the wild-type human H1N1 strain. The reassortant viruses in this research were used as a model to study the early stage of evolution of avian-human reassortant influenza viruses. We showed that the RNP complex with the PB1 gene from A2093-H9N2 in the background of the WSN-H1N1 virus significantly promoted the activity of RNPs complex in a Dual-Luciferase Assay System. In MDCK cells, the PB1-reassortant virus was found to replicate with similar efficiency as wt WSN-H1N1 virus. In DF1 cells, the replication of the PB1-reassortant was reduced compared to wt WSN-H1N1 virus. The other reassortant viruses replicated less efficiently in both MDCK and DF1 cells compared to wt WSN-H1N1 virus. The reassortant viruses were able to replicate efficiently in the mouse turbinate and lung, to similar levels as wt WSN-H1N1 virus. Except for the PB2-reassortant virus that showed significantly reduced replication in the mouse lung. The body weight of the mice was measured as an indication for virulence (replication ability) of the viruses. Most interestingly, we observed increased virulence (replication ability) in mice for the PB1-reassortant virus. Due to the low pathogenicity of the virus, no mortality other than decrease in body weight were observed in the infected mice. A previous study detected a high polymerase activity of the combination of mammalian PB2 gene and avian PB1 gene in human cells [61]. The involvement of avian PB1 gene in mammalian-adapted virus might obtain a higher virulence in new host by generating adaptive mutations under a new selection pressure. To obtain more information on the replication and evolution of the WSN-H1N1 virus containing inner gene segments of the avian H9N2 virus, we performed serial passaging of the reassortant viruses in MDCK cells and in mice.

In this study, the “mutation rate” was calculated during serial passaging of the reassortant viruses. The mutation rate is therefore a combination of initial errors made during RNA replication, combined with the effects of host selection [62]. Mutations in HA may lead to changes of antibody or receptor binding, and may be preferentially selected [63, 64]. For all reassortant viruses in this study, we observed a higher mutation rate (mutation/site/infection cycle) in mouse lungs compared to MDCK cells. This difference was likely caused by the increased selection pressure mediated by the mouse immune system. However, the absolute mutation rate measured for the viruses may differ dependent on the host species. The mutation rate measured on HA gene was more than two-fold increased for the PA-reassortant virus compared to wt WSN-H1N1 and A2093-H9N2 viruses, and more than 1.5 times for the PB1-reassortant virus. Previous studies suggested the mutation rate of influenza A viruses ranged from 7.1 × 10^−6^ to 4.5 × 10^−5^ substitutions per nucleotide per cell infection cycle (s/n/c) of the whole genome [65, 66]. In this study, we measured mutation rates of 5.0 × 10^−4^ and 6.2 × 10^−4^ mutation/site/infection cycle on HA gene for the PB1 and PA-reassortant virus, which is higher than previously reported. We showed that polymerase activity was increased for the PB1-reassortant virus, which may have resulted in an increased error-rate during RNA replication. However, decreased polymerase activity was measured for the PA-reassortant. The higher mutation rate observed therefore may also result from the strong selection pressure on the reassortant viruses due to their novel genetic composition. We analyzed the HA sequence in this study, and higher selection pressure may be expected for virus surface protein [67]. Furthermore, the mutation rate may not only depend on the gene segment analyzed, but also on the virus subtype as was reported previously [67, 68]. Finally, differences in the analysis methods may have contributed to variation in the error-rates reported [69, 70]. The number/ratio of non-synonymous mutations is indicator for the selection pressure on the virus [71]. With similar high SBS numbers, the PB1-reassortant virus showed the lowest precentages of non-synonymous changes, whereas in PA-reassortant virus only non-synonymous changes were found. This high percentage of non-synonymous mutations in PA-reassortant virus was also reflected in a high relative genetic distance, suggesting there is a strong positive selection on the PA-reassortant virus. The serial passaging experiments, in which five host-infection circles were observed, showed that the substitution rates of both the PA and PB1-reassortant viruses were increased compared to the other reassortant viruses. This suggests that reassortant virusses obtaining the PB1 gene from avian H9N2 are more likely to rapidly adapt to new hosts. This in accordance with a previous study which showed that virus replication was more efficient when PB1 was derived from an avian virus, regardless of the origin of the other proteins [72]. Furthermore, we identified a mutation hot-spot in the HA-gene, that is located near the antigenic and receptor binding sites [73–75]. We measured a significantly increased substitution frequency for the 350–950 domain of HA1 compared to the complete ORF region. Our results are consistent with previous studies which showed that the head domain of HA evolves faster than the stalk domain [76]. This domain included the 130 helix and 220 loop structure of HA head which are exposed to the surface and therefore can be easily captured by host immune system [77]. Together with the receptor-binding function, the highly mutable HA1 domain might compromise viral replicative fitness, which means the globular head of HA are highly tolerant of mutations [78]. We further indicated that the mutation patterns could be highly influenced by the reassortant viral vRdRp complex, especially in reassortment between human and avian viruses. However, further research will be required to provide more insight in the intracellular mechanism at molecular level.

Reassortment events between human and avian influenza viruses in combination with rapid evolution and adaptation due to error-prone replication may lead to a novel human pandemic. The H9N2 virus is currently the most frequently detected subtype (particularly in live bird markets) and has become endemic in poultry across Asia since 1990s [79]. Several studies provided evidence of interspecies transmission of H9N2 virus from poultry to mammals, such as swine [80, 81]. Swine may represent “mixing vessel” for influenza viruses as they are susceptible for infected with swine, human and avian influenza viruses [82]. An experimental study showed the replication of H9N2 virus (A/guinea fowl/Hong Kong/WF10/99, A/guinea fowl/Hong Kong/NT184/03) in mice without adaptation [83], likely because of its properties of internal genes related to polymerase function. As human infections with avian H9N2 viruses have been reported [8, 14], there is a high probability of reassortment with human influenza viruses.

## Conclusions

In this study, we showed that reassortment between a human H1N1 virus and the avian H9N2 virus might potentially result in a novel virus that can readily adapt to humans: the reassortant virus with the avian PB1 gene showed increased polymerase activity, better replication in mouse lung and high mutation rate at HA gene, in particular in the HA1 domain related to receptor binding and immunogenicity. Therefore, human infections with avian H9N2 viruses and the possible emergence of reassortant influenza viruses carrying avian H9N2 polymerase genes must be carefully monitored for pandemic preparedness.

## Supplementary Information

**Additional file 1: Fig. S1**. The matrix of scatterplots for F_g/nt_ of different HA domains. The matrix was visualized in R. Diagonal boxes indicated the different HA domains. Only the scatterplots (a and c) of “ORF” and “HotSpot” displayed linear relationship.

**Additional file 2: Table S1**. Specific indexes (Barcodes) for reassortant viruses from different hosts. **Table S2** Primer sequence for segment amplification of HA gene. **Table S3** Total substitution frequency (F_i_) at every location at HA ORF region. **Table S4** The summary of mutation information. Only the locations with Fi > 0.01 were listed. **Table S5** The genetic distances of HA from reassortant viruses and WSN strain.

## Data Availability

All data generated or analysed during this study are included in this published article [and its Additional files 1 and 2:].

## Abbreviations

PB2: Polymerase basic protein 2
PB1: Polymerase basic protein 1
PA: Polymerase acidic protein
NP: RNA-binding nucleoprotein
HA: Hemagglutinin
NA: Neuraminidase
NP: Nucleoprotein
RNP: Ribonucleoprotein
NGS: Next-Generation Sequencing
HPAIV: Highly pathogenicity avian influenza virus
LPAIV: Low pathogenicity avian influenza viruses
AIV: Avian influenza virus
SA: Sialic acid
BSL-2: Biosafety level 2
MDCK: Madin-Darby Canine Kidney
DMEM: Dulbecco's modified Eagle's medium
FBS: Fetal bovine serum
SPF: Specific-pathogen-free
ORF: Open reading frame
RLU: Relative light unit
MOI: Multiplicity of infection
SFM: Serum-Free Media
PFU: Plaque-forming units
PBS: Phosphate-buffered saline
SBS: Single base substitutions

## References

[1] Perez DR, Lim W, Seiler JP, Yi G, Peiris M, Shortridge KF, Webster RG (2003). Role of quail in the interspecies transmission of H9 influenza A viruses: molecular changes on HA that correspond to adaptation from ducks to chickens. J Virol.

[2] Lee CW, Song CS, Lee YJ, Mo IP, Garcia M, Suarez DL, Kim SJ (2000). Sequence analysis of the hemagglutinin gene of H9N2 Korean avian influenza viruses and assessment of the pathogenic potential of isolate MS96. Avian Dis.

[3] Perk S, Panshin A, Shihmanter E, Gissin I, Pokamunski S, Pirak M, Lipkind M, Schudel A, Lombard M (2006). Ecology and molecular epidemiology of H9N2 avian influenza viruses isolated in Israel during 2000–2004 epizootic. Dev Biol (Basel).

[4] Lee YJ, Shin JY, Song MS, Lee YM, Choi JG, Lee EK, Jeong OM, Sung HW, Kim JH, Kwon YK (2007). Continuing evolution of H9 influenza viruses in Korean poultry. Virology.

[5] Liu JH, Okazaki K, Mweene A, Shi WM, Wu QM, Su JL, Zhang GZ, Bai GR, Kida H (2004). Genetic conservation of hemagglutinin gene of H9 influenza virus in chicken population in Mainland China. Virus Genes.

[6] Kawaoka Y, Chambers TM, Sladen WL, Webster RG (1988). Is the gene pool of influenza viruses in shorebirds and gulls different from that in wild ducks?. Virology.

[7] Jackwood MW, Stallknecht DE (2007). Molecular epidemiologic studies on North American H9 avian influenza virus isolates from waterfowl and shorebirds. Avian Dis.

[8] Butt K, Smith GJ, Chen H, Zhang L, Leung YC, Xu K, Lim W, Webster RG, Yuen K, Peiris JM (2005). Human infection with an avian H9N2 influenza A virus in Hong Kong in 2003. J Clin Microbiol.

[9] Peiris M, Yuen K, Leung C, Chan K, Ip P, Lai R, Orr W, Shortridge K (1999). Human infection with influenza H9N2. The Lancet.

[10] Chakraborty A, Arifeen S, Streafield P (2011). Outbreak of mild respiratory disease caused by H5N1 and H9N2 infections among young children in Dhaka, Bangladesh, 2011. Health Sci Bull.

[11] Ali M, Yaqub T, Mukhtar N, Imran M, Ghafoor A, Shahid MF, Naeem M, Iqbal M, Smith GJ, Su YC (2019). Avian influenza A (H9N2) virus in poultry worker, Pakistan, 2015. Emerg Infect Dis.

[12] Alexander DJ (2007). Summary of avian influenza activity in Europe, Asia, Africa, and Australasia, 2002–2006 (Resumen de la actividad de la influenza aviar en Europa, Asia, África y Australasia entre los años 2002–2006). Avian Dis.

[13] Guo Y, Krauss S, Senne D, Mo I, Lo K, Xiong X, Norwood M, Shortridge K, Webster R, Guan Y (2000). Characterization of the pathogenicity of members of the newly established H9N2 influenza virus lineages in Asia. Virology.

[14] Guo Y, Li J, Cheng X (1999). Discovery of men infected by avian influenza A (H9N2) virus. Chin J Exp Clin Virol.

[15] Peacock TTP, James J, Sealy JE, Iqbal M (2019). A global perspective on H9N2 avian influenza virus. Viruses.

[16] Potdar V, Hinge D, Satav A, Simões EA, Yadav PD, Chadha MS (2019). Laboratory-confirmed avian influenza A (H9N2) virus infection, India, 2019. Emerg Infect Dis.

[17] Influenza at the human-animal interface https://www.who.int/influenza/human_animal_interface/HAI_Risk_Assessment/en/

[18] Peacock TP, Benton DJ, Sadeyen J-R, Chang P, Sealy JE, Bryant JE, Martin SR, Shelton H, McCauley JW, Barclay WS (2017). Variability in H9N2 haemagglutinin receptor-binding preference and the pH of fusion. Emerg Microbes Infect.

[19] Huang Y, Li X, Zhang H, Chen B, Jiang Y, Yang L, Zhu W, Hu S, Zhou S, Tang Y (2015). Human infection with an avian influenza A (H9N2) virus in the middle region of China. J Med Virol.

[20] Zhou L, Chen E, Bao C, Xiang N, Wu J, Wu S, Shi J, Wang X, Zheng Y, Zhang Y (2018). Clusters of human infection and human-to-human transmission of avian influenza A (H7N9) virus, 2013–2017. Emerg Infect Dis.

[21] Guan Y, Shortridge KF, Krauss S, Chin PS, Dyrting KC, Ellis TM, Webster RG, Peiris M (2000). H9N2 influenza viruses possessing H5N1-like internal genomes continue to circulate in poultry in Southeastern China. J Virol.

[22] Li X, Sun J, Lv X, Wang Y, Li Y, Li M, Liu W, Zhi M, Yang X, Fu T (2020). Novel reassortant avian influenza A (H9N2) virus isolate in migratory waterfowl in Hubei province, China. Front Microbiol.

[23] Kimble JB, Sorrell E, Shao H, Martin PL, Perez DR (2011). Compatibility of H9N2 avian influenza surface genes and 2009 pandemic H1N1 internal genes for transmission in the ferret model. Proc Natl Acad Sci.

[24] Guan Y, Shortridge KF, Krauss S, Webster RG (1999). Molecular characterization of H9N2 influenza viruses: were they the donors of the “internal” genes of H5N1 viruses in Hong Kong?. Proc Natl Acad Sci.

[25] White MC, Lowen AC (2018). Implications of segment mismatch for influenza A virus evolution. J Gen Virol.

[26] Sun Y, Qin K, Wang J, Pu J, Tang Q, Hu Y, Bi Y, Zhao X, Yang H, Shu Y (2011). High genetic compatibility and increased pathogenicity of reassortants derived from avian H9N2 and pandemic H1N1/2009 influenza viruses. Proc Natl Acad Sci.

[27] Scholtissek C (1995). Molecular evolution of influenza viruses. Virus Genes.

[28] Ince WL, Gueyembaye A, Bennink JR, Yewdell JW (2013). Reassortment complements spontaneous mutation in influenza A virus NP and M1 genes to accelerate adaptation to a new host. J Virol.

[29] Holmes EC, Ghedin E, Miller N, Taylor J, Bao Y, George KS, Grenfell BT, Salzberg SL, Fraser CM, Lipman DJ (2005). Whole-Genome Analysis of Human Influenza A Virus Reveals Multiple Persistent Lineages and Reassortment among Recent H3N2 Viruses. PLOS Biol.

[30] Sorrell EM, Wan H, Araya Y, Song H, Perez DR, Palese P (2009). Minimal molecular constraints for respiratory droplet transmission of an avian-human H9N2 influenza A virus. Proc Natl Acad Sci USA.

[31] Lam TT, Zhou B, Wang J, Chai Y, Shen Y, Chen X, Ma C, Hong W, Chen Y, Zhang Y (2015). Dissemination, divergence and establishment of H7N9 influenza viruses in China. Nature.

[32] Gu M, Chen H, Li Q, Huang J, Zhao M, Gu X, Jiang K, Wang X, Peng D, Liu X (2014). Enzootic genotype S of H9N2 avian influenza viruses donates internal genes to emerging zoonotic influenza viruses in China. Vet Microbiol.

[33] Hao X, Wang X, Hu J, Gu M, Wang J, Deng Y, Jiang D, He D, Xu H, Yang Y (2019). The PB2 and M genes of genotype S H9N2 virus contribute to the enhanced fitness of H5Nx and H7N9 avian influenza viruses in chickens. Virology.

[34] Best SM, Kerr PJ (2000). Coevolution of host and virus: the pathogenesis of virulent and attenuated strains of myxoma virus in resistant and susceptible european rabbits. Virology.

[35] Li X, Shi J, Guo J, Deng G, Zhang Q, Wang J, He X, Wang K, Chen J, Li Y (2014). Genetics, receptor binding property, and transmissibility in mammals of naturally isolated H9N2 avian influenza viruses. PLOS Pathogens.

[36] Lindstrom SE, Cox NJ, Klimov A (2004). Genetic analysis of human H2N2 and early H3N2 influenza viruses, 1957–1972: evidence for genetic divergence and multiple reassortment events. Virology.

[37] Fodor E (2013). The RNA polymerase of influenza a virus: mechanisms of viral transcription and replication. Acta Virol.

[38] Biswas SK, Boutz PL, Nayak DP (1998). Influenza virus nucleoprotein interacts with influenza virus polymerase proteins. J Virol.

[39] Andino R, Rieckhof GE, Achacoso PL, Baltimore D (1993). Poliovirus RNA synthesis utilizes an RNP complex formed around the 5′-end of viral RNA. EMBO J.

[40] de la Luna S, Martín J, Portela A, Ortín J (1993). Influenza virus naked RNA can be expressed upon transfection into cells co-expressing the three subunits of the polymerase and the nucleoprotein from simian virus 40 recombinant viruses. J Gen Virol.

[41] Watanabe T, Watanabe S, Shinya K, Kim JH, Hatta M, Kawaoka Y (2009). Viral RNA polymerase complex promotes optimal growth of 1918 virus in the lower respiratory tract of ferrets. Proc Natl Acad Sci.

[42] Graef KM, Vreede FT, Lau Y-F, McCall AW, Carr SM, Subbarao K, Fodor E (2010). The PB2 subunit of the influenza virus RNA polymerase affects virulence by interacting with the mitochondrial antiviral signaling protein and inhibiting expression of beta interferon. J Virol.

[43] Hatta M, Gao P, Halfmann P, Kawaoka Y (2001). Molecular basis for high virulence of Hong Kong H5N1 influenza A viruses. Science.

[44] Labadie K, Afonso EDS, Rameix-Welti M-A, Van Der Werf S, Naffakh N (2007). Host-range determinants on the PB2 protein of influenza A viruses control the interaction between the viral polymerase and nucleoprotein in human cells. Virology.

[45] Shinya K, Hamm S, Hatta M, Ito H, Ito T, Kawaoka Y (2004). PB2 amino acid at position 627 affects replicative efficiency, but not cell tropism, of Hong Kong H5N1 influenza A viruses in mice. Virology.

[46] Pappas C, Aguilar PV, Basler CF, Solórzano A, Zeng H, Perrone LA, Palese P, García-Sastre A, Katz JM, Tumpey TM (2008). Single gene reassortants identify a critical role for PB1, HA, and NA in the high virulence of the 1918 pandemic influenza virus. Proc Natl Acad Sci.

[47] Teng Q, Xu D, Shen W, Liu Q, Rong G, Li X, Yan L, Yang J, Chen H, Hai Y (2016). A single mutation at position 190 in hemagglutinin enhances binding affinity for human type sialic acid receptor and replication of H9N2 avian influenza virus in mice. J Virol.

[48] Hoffmann E, Neumann G, Kawaoka Y, Hobom G, Webster RG (2000). A DNA transfection system for generation of influenza A virus from eight plasmids. Proc Natl Acad Sci USA.

[49] Hatta M, Kawaoka Y (2001). Molecular basis for high virulence of Hong Kong H5N1 influenza A viruses. Science.

[50] Tobita K, Sugiura A, Enomoto C, Furuyama M (1975). Plaque assay and primary isolation of influenza A viruses in an established line of canine kidney cells (MDCK) in the presence of trypsin. Med Microbiol Immunol.

[51] Morelli MJ, Wright CF, Knowles NJ, Juleff N, Paton DJ, King DP, Haydon DT (2013). Evolution of foot-and-mouth disease virus intra-sample sequence diversity during serial transmission in bovine hosts. Vet Res.

[52] Team RC (2013) R: A language and environment for statistical computing

[53] Van den Hoecke S, Verhelst J, Vuylsteke M, Saelens X (2015). Analysis of the genetic diversity of influenza A viruses using next-generation DNA sequencing. BMC Genomics.

[54] Yoshikawa T, Matsuo K, Matsuo K, Suzuki Y, Nomoto A, Tamura S-I, Kurata T, Sata T (2004). Total viral genome copies and virus–Ig complexes after infection with influenza virus in the nasal secretions of immunized mice. J Gen Virol.

[55] Worobey M, Han G-Z, Rambaut A (2014). Genesis and pathogenesis of the 1918 pandemic H1N1 influenza A virus. Proc Natl Acad Sci.

[56] Kilbourne ED (2006). Influenza pandemics of the 20th century. Emerg Infect Dis.

[57] Garten RJ, Davis CT, Russell CA, Shu B, Lindstrom S, Balish A, Sessions WM, Xu X, Skepner E, Deyde V (2009). Antigenic and genetic characteristics of swine-origin 2009 A (H1N1) influenza viruses circulating in humans. Science.

[58] Smith GJ, Vijaykrishna D, Bahl J, Lycett SJ, Worobey M, Pybus OG, Ma SK, Cheung CL, Raghwani J, Bhatt S (2009). Origins and evolutionary genomics of the 2009 swine-origin H1N1 influenza A epidemic. Nature.

[59] Teng Q, Xu D, Shen W, Liu Q, Rong G, Li X, Yan L, Yang J, Chen H, Yu H (2016). A single mutation at position 190 in hemagglutinin enhances binding affinity for human type sialic acid receptor and replication of H9N2 avian influenza virus in mice. J Virol.

[60] Yang J, Huang M, Qiao S, Zhang P, Teng Q, Li X, Liu Q, Chen H, Zhang Z, Yan D, Li Z (2021). Replication and virulence of chimeric bat influenza viruses in mammalian and avian cells and in mice. Microb Pathog.

[61] Li OT, Chan MC, Leung CS, Chan RW, Guan Y, Nicholls JM, Poon LL (2009). Full factorial analysis of mammalian and avian influenza polymerase subunits suggests a role of an efficient polymerase for virus adaptation. PLOS ONE.

[62] Sanjuán R, Domingo-Calap P (2016). Mechanisms of viral mutation. Cell Mol Life Sci.

[63] Hay AJ, Gregory V, Douglas AR, Lin YP (2001). The evolution of human influenza viruses. Philos Trans R Soc Lond B Biol Sci.

[64] Suzuki Y, Gojobori T (1999). A method for detecting positive selection at single amino acid sites. Mol Biol Evol.

[65] Sanjuán R, Nebot MR, Chirico N, Mansky LM, Belshaw R (2010). Viral mutation rates. J Virol.

[66] Pauly MD, Procario M, Lauring AS: The mutation rates and mutational bias of influenza A virus. bioRxiv 2017:110197

[67] Webster RG, Bean WJ, Gorman OT, Chambers TM, Kawaoka Y (1992). Evolution and ecology of influenza A viruses. Microbiol Mol Biol Rev.

[68] Nobusawa E, Sato K (2006). Comparison of the mutation rates of human influenza A and B viruses. J Virol.

[69] Pauly MD, Procario MC, Lauring AS (2017). A novel twelve class fluctuation test reveals higher than expected mutation rates for influenza A viruses. Elife.

[70] Parvin JD, Moscona A, Pan W, Leider J, Palese P (1986). Measurement of the mutation rates of animal viruses: influenza A virus and poliovirus type 1. J Virol.

[71] Hu T, Banzhaf W (2008) Nonsynonymous to synonymous substitution ratio Ka/ks: measurement for rate of evolution in evolutionary computation. In: International conference on parallel problem solving from nature. Springer, pp 448–457

[72] Naffakh N, Massin P, Escriou N, Crescenzo-Chaigne B, van der Werf S (2000). Genetic analysis of the compatibility between polymerase proteins from human and avian strains of influenza A viruses. Microbiology.

[73] Ha Y, Stevens DJ, Skehel JJ, Wiley DC (2001). X-ray structures of H5 avian and H9 swine influenza virus hemagglutinins bound to avian and human receptor analogs. Proc Natl Acad Sci.

[74] Wan Z, Ye J, Xu L, Shao H, Jin W, Qian K, Wan H, Qin A (2014). Antigenic mapping of the hemagglutinin of an H9N2 avian influenza virus reveals novel critical amino acid positions in antigenic sites. J Virol.

[75] Peacock T, Reddy K, James J, Adamiak B, Barclay W, Shelton H, Iqbal M (2016). Antigenic mapping of an H9N2 avian influenza virus reveals two discrete antigenic sites and a novel mechanism of immune escape. Sci Rep.

[76] Kirkpatrick E, Qiu X, Wilson PC, Bahl J, Krammer F (2018). The influenza virus hemagglutinin head evolves faster than the stalk domain. Sci Rep.

[77] Raymond DD, Bajic G, Ferdman J, Suphaphiphat P, Settembre EC, Moody MA, Schmidt AG, Harrison SC (2018). Conserved epitope on influenza-virus hemagglutinin head defined by a vaccine-induced antibody. Proc Natl Acad Sci.

[78] Doud MB, Bloom JD (2016). Accurate measurement of the effects of all amino-acid mutations on influenza hemagglutinin. Viruses.

[79] Alexander DJ (2007). An overview of the epidemiology of avian influenza. Vaccine.

[80] Cong Y-L, Wang C-F, Yan C-M, Peng J-S, Jiang Z-L, Liu J-H (2008). Swine infection with H9N2 influenza viruses in China in 2004. Virus Genes.

[81] Yu H, Hua R-H, Wei T-C, Zhou Y-J, Tian Z-J, Li G-X, Liu T-Q, Tong G-Z (2008). Isolation and genetic characterization of avian origin H9N2 influenza viruses from pigs in China. Vet Microbiol.

[82] Ma W, Kahn RE, Richt JA (2009). The pig as a mixing vessel for influenza viruses: human and veterinary implications. J Mol Genet Med.

[83] Choi YK, Ozaki H, Webby RJ, Webster RG, Peiris JS, Poon L, Butt C, Leung YHC, Guan Y (2004). Continuing evolution of H9N2 influenza viruses in Southeastern China. J Virol.

