# Supplementary figures and images for: The PB1 gene from H9N2 avian influenza virus showed high compatibility and increased mutation rate after reassorting with a human H1N1 influenza virus
Source: Virol J. 2022 Jan 25;19:20. doi: 10.1186/s12985-022-01745-x (PMC8788113; doi:10.1186/s12985-022-01745-x)

## Slide 1
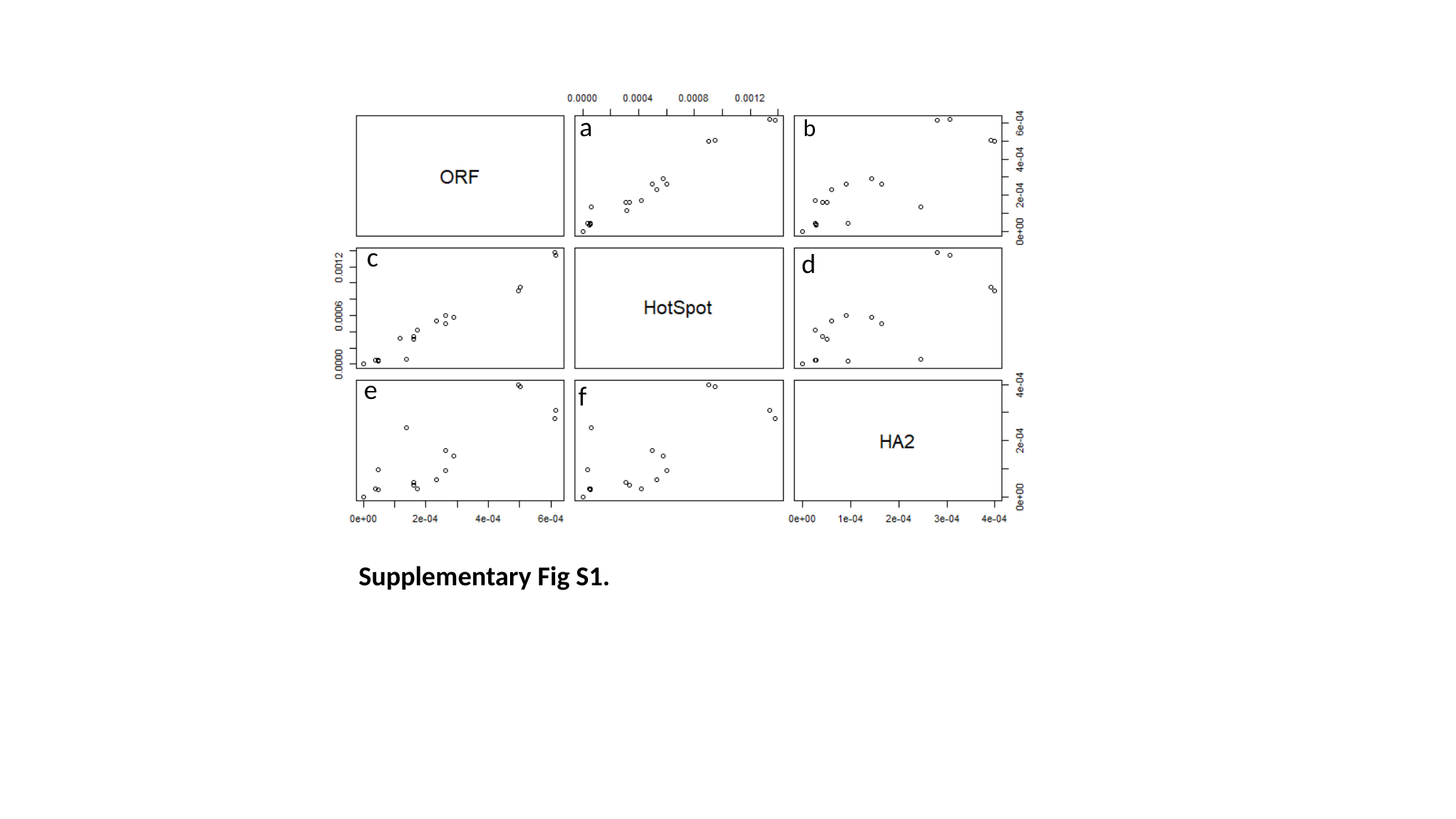

a
b
c
d
e
f
Supplementary Fig S1.

Supplement: Supplementary file 1 — Additional file 1: Fig. S1. The matrix of scatterplots for Fg/nt of different HA domains. The matrix was visualized in R. Diagonal boxes indicated the different HA domains. Only the scatterplots (a and c) of “ORF” and “HotSpot” displayed linear relationship. [file 12985_2022_1745_MOESM1_ESM.pptx]
